# DNA hydroxymethylation combined with carotid plaques as a novel biomarker for coronary atherosclerosis

**DOI:** 10.18632/aging.101972

**Published:** 2019-05-23

**Authors:** Dan Jiang, Ying Wang, Guanglei Chang, Qin Duan, Linna You, Min Sun, Chunxiao Hu, Lei Gao, Shiyong Wu, Hongmei Tao, Kai Lu, Dongying Zhang

**Affiliations:** 1 Department of Cardiology, The First Affiliated Hospital of Chongqing Medical University, Yuzhong, Chongqing, China; 2 Laboratory Research Center, The First Affiliated Hospital of Chongqing Medical University, Yuzhong, Chongqing, China; 3 Department of Cardiology, The First Branch of the First Affiliated Hospital of Chongqing Medical University, Yuzhong, Chongqing, China; * Equal contribution

**Keywords:** methylation, hydroxymethylation, carotid plaque, coronary atherosclerosis

## Abstract

Little is known about the diagnostic value of DNA methylation and hydroxymethylation for coronary atherosclerosis. Carotid plaque is a common marker for coronary atherosclerosis. Our aim is to determine whether DNA methylation and hydroxymethylation combined with carotid plaques can be useful to the diagnosis of coronary atherosclerosis. The 5-methylcytosine (5-mC) and 5-hydroxymethylcytosine (5-hmC) levels from peripheral blood mononuclear cells (PBMCs) were measured in 113 enrolled patients. Crouse score and Gensini score were used to evaluate the severity of carotid and coronary atherosclerosis, respectively. With the increasing of severity of carotid plaque, a stepwise upward trend was observed in 5-mC and 5-hmC levels from PBMCs, which were significantly correlated with the risk factors, Crouse score and Gensini score. Crouse score and 5-hmC, not 5-mC, were the risk factors for coronary atherosclerosis after adjustment for the risk factors (the history of diabetes, FPG and HbA1c). Receiver operating characteristic (ROC) analysis indicated that 5-hmC combined with Crouse score was the diagnostic biomarker for coronary atherosclerosis, with the highest areas under the curve (AUC) for 0.980 (0.933–0.997), valuable sensitivity for 96.23% and specificity for 91.67%. These findings suggest 5-hmC level combined with Crouse score may provide the meaningful information for coronary atherosclerosis diagnosis.

## INTRODUCTION

In recent years, the role of epigenetics in atherosclerosis (AS) has become a topic of intense research interest, especially DNA methylation and hydroxymethylation modification [1–3]. DNA methylation is the conversion process of cytosine (C) to 5-methylcytosine (5-mC) catalyzed by DNA methyltransferase (DNMTs), while DNA hydroxymethylation is the oxidation process of 5-mC to 5-hydroxymethylcytosine (5-hmC) in the presence of ten-eleven translocation (TETs) [4, 5]. 5-mC and 5-hmC are usually used to evaluate global methylation and hydroxymethylation levels. Increasing studies have focused on the association between DNA methylation and hydroxymethylation and the development of AS, especially carotid atherosclerosis (CAS) [6, 7] and coronary atherosclerosis [8–10]. However, little is known about the diagnostic value of DNA methylation and hydroxymethylation for coronary atherosclerosis.

Carotid artery is superficially fixed due to the location of the artery and easy to identify by ultrasound, often used to evaluate the severity of systemic atherosclerosis which lead to occlusion of blood vessels, myocardial infarction, peripheral vascular disease and stroke [11, 12]. As common indicators of carotid ultrasound, carotid intima-media thickness (CIMT) thickening is an early change in CAS and plaque formation is a typical marker of CAS. Carotid atherosclerosis has a significant correlation with coronary atherosclerosis, usually earlier than coronary atherosclerosis [13]. The plaque morphology and their anatomical location at branching points is similar in the carotid and coronary arterial systems [14, 15]. As a screening method for coronary atherosclerosis, carotid plaque in asymptomatic elderly patients have a strong predictive effect on morbidity and mortality of cardiovascular disease and the degree and plaque type of coronary atherosclerosis [16–21].

However, the clinical diagnostic value of carotid plaque for coronary atherosclerosis is relatively limited. Therefore, we aim to determine whether DNA methylation and hydroxymethylation combined with carotid plaques can be useful to the diagnosis of coronary atherosclerosis.

## RESULTS

### Baseline characteristics of CAS patients and control subjects

Baseline characteristics of different degrees of CAS patients and control subjects were shown in Table 1. 91 CAS patients included 53 patients with coronary heart disease (CHD) and 11 patients with stroke events. All CAS patients were divided into three groups according to the tertiles of Crouse score, including bottom tertile (n=31), middle tertile (n=30) and top tertile (n=30). Gender, age, smoker, history of hypertension and diabetes, BMI were well matched among all groups. Except FPG, no statistical difference was found in HbA1c, TC, TG, LDL-c, HDL-c and hs-CRP levels among control and three CAS groups. With the increasing of severity of carotid plaque and Crouse score, a stepwise upward trend was observed in CIMT and Gensini score. More importantly, the middle tertile and top tertile had higher Gensini score than the control group and bottom tertile, respectively.

**Table 1:** Baseline characteristics of different degrees of carotid atherosclerosis.

**CAS patients (Crouse score, n=91)**					
**Characteristics**	**Control (n=22)**	**Bottom tertile (n=31)**	**Middle tertile (n=30)**	**Top tertile (n=30)**	***P* value**
Male/Female	9/13	17/14	19/11	19/11	0.348
Age (years)	72.77±4.84	73.39±5.77	72.77±4.55	73.07±5.66	0.965
Smoker (n)	4 (18.2%)	10 (32.3%)	12 (40.0%)	15 (50.0%)	0.118
History of HP (n)	12 (54.5%)	16 (51.6%)	22 (73.3%)	23 (76.7%)	0.105
History of DM (n)	5 (22.7%)	11 (35.5%)	13 (43.3%)	13 (43.3%)	0.413
CHD (n)	0	12 (38.7%)^*^	17 (56.7%)^*^	24 (80.0%)^*#^	<0.001
Stroke (n)	1 (4.5%)	1 (3.2%)	4 (13.3%)	6 (20.0%)	0.147
BMI (Kg/m2)	24.70±3.64	24.51±2.83	24.13±2.78	24.36±3.39	0.924
FPG (mmol/L)	5.20 (4.95–5.50)	5.40 (5.10–6.00)	5.90 (5.43–7.5) ^*^	5.50 (5.20–6.23)	0.020
HbA1c (%)	6.00 (5.70–6.20)	5.90 (5.60–6.30)	6.20 (5.68–6.93)	6.00 (5.70–7.15)	0.673
TC (mmol/L)	3.09 (1.18–4.58)	3.36 (2.24–4.86)	3.91 (3.12–4.31)	3.93 (2.90–4.74)	0.439
TG (mmol/L)	1.17 (0.77–1.74)	1.16 (0.86–1.92)	1.40 (0.94–1.99)	1.42 (1.00–2.26)	0.227
LDL-c (mmol/L)	2.21±1.08	2.30±1.08	2.53±0.86	2.24±1.02	0.843
HDL-c (mmol/L)	1.63 (1.30–2.78)	1.22 (1.04–1.99)	1.22 (1.05–1.50)	1.34 (0.94–2.39)	0.151
hs-CRP (mg/L)	1.02 (0.33–3.47)	1.49 (0.59–4.08)	1.33 (1.04–4.78)	1.08 (0.56–5.35)	0.542
CIMT (mm)	0.75 (0.65–0.85)	0.75 (0.70–0.85)	0.80 (0.75–0.90)	0.85 (0.75–1.00)^*^	0.014
Crouse score	0	1.90 (1.60–2.60)^*^	4.20 (3.60–4.90)^*#^	6.75 (6.38–7.63)^*#^	<0.001
Gensini score	0	0 (0–57.0)	20.0 (0–86.1)^*#^	42.8 (20.4–74.5)^*#^	<0.001
5-mC (%)	2.81 (2.04–3.39)	3.09 (2.64–3.75)	3.40 (2.96–4.21)^*^	3.86 (3.34–4.90)^*#^	<0.001
5-hmC (%)	0.06 (0.03–0.09)	0.08 (0.04–0.19)	0.17 (0.09–0.30)^*^	0.22 (0.12–0.30)^*#^	<0.001

Data are presented as mean ± SD, median (interquartile ranges), or number (%). HP, hypertension; DM, diabetes mellitus; CHD, coronary heart disease; BMI, body mass index; FPG, fasting plasma glucose; HbA1c, glycosylated hemoglobin; TC, total cholesterol; TG, triglycerides; LDL-c, low-density lipoprotein cholesterol; HDL-c, high-density lipoprotein cholesterol; hs-CRP, high sensitivity C reactive protein; CIMT, carotid intima media thickness. ^*^ P<0.05 comparing with control group; ^#^ P<0.05 comparing with bottom tertile group.

### DNA methylation and hydroxymethylation levels of CAS patients and control subjects

**Figure 1:**
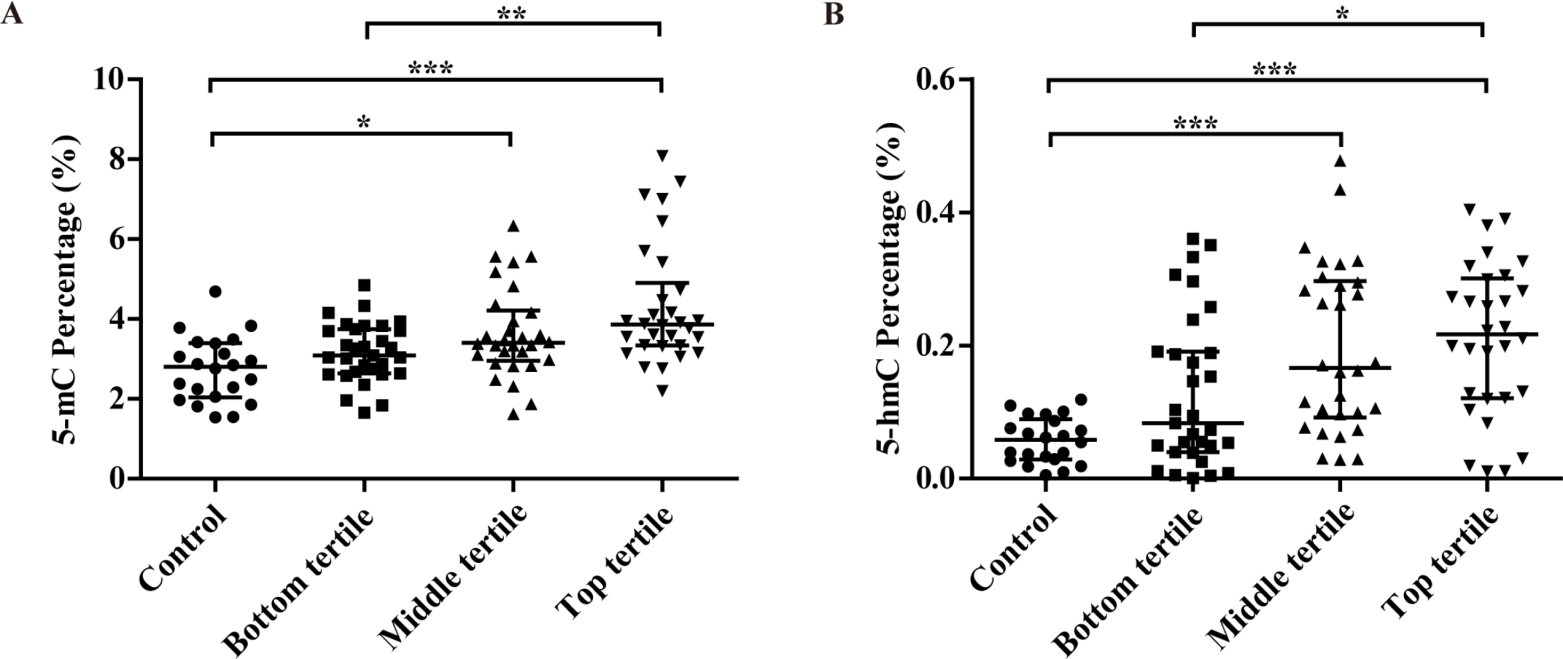
Associations between the percentage of 5-mC (**A**), 5-hmC (**B**) and the severity of carotid atherosclerosis in CAS patients (evaluated by plaque thickness). ^*^ *P*<0.05; ^**^ *P*<0.01; ^***^ *P*<0.001.

As shown in Figure 1, with the increasing of severity of carotid plaque and Crouse score, a stepwise upward trend was observed in the 5-mC level. 5-mC percentage of four groups were 2.81 (2.04–3.39)%, 3.09 (2.64–3.75)%, 3.40 (2.96–4.21)% and 3.86 (3.34–4.90)%, respectively. The middle and top tertile had significantly higher 5-mC level than control group.

5-hmC level had significant difference among four groups with the increasing of severity of carotid plaque and Crouse score, 0.06 (0.03–0.09)% for control group, 0.08 (0.04–0.19)% for the bottom tertile, 0.17 (0.09–0.30)% for the middle tertile and 0.22 (0.12–0.30)% for the top tertile. The 5-hmC level in three CAS groups was more than several times (once, twice and three, respectively) higher than that in control group.

### Correlation analysis of 5-mC, 5-hmC and Gensini score with selected covariates

Spearman correlation coefficients between 5-mC and 5-hmC levels and selected cardiovascular risk factors were shown in Table 2. 5-mC and 5-hmC levels were both positively correlated with FPG, while HbA1c and HDL-c were only significantly associated with 5-hmC. It was shown that 5-mC and 5-hmC levels were positively associated with Crouse score.

**Table 2:** Spearman correlation coefficients of the percentage of 5-mC, 5-hmC and Gensini score with selected covariates.

**Variable**	**5-mC (%)**		**5-hmC (%)**		**Gensini score**	
	**r**	***P***	**r**	***P***	**r**	***P***
FPG (mmol/L)	0.260	0.005	0.343	<0.001	0.356	<0.001
HbA1c (%)	0.149	0.116	0.189	0.045	0.250	0.007
TC (mmol/L)	–0.083	0.385	0.030	0.751	0.113	0.234
TG (mmol/L)	0.226	0.016	0.176	0.066	0.265	0.005
LDL-c (mmol/L)	0.002	0.981	0.059	0.531	0.173	0.067
HDL-c (mmol/L)	–0.096	0.312	–0.246	0.009	–0.322	0.001
hs-CRP (mg/L)	0.122	0.198	0.181	0.055	0.179	0.058
CIMT (mm)	0.125	0.188	0.115	0.224	0.162	0.087
Crouse score	0.471	<0.001	0.509	<0.001	0.546	<0.001
5-mC (%)	-	-	0.579	<0.001	0.469	<0.001
5-hmC (%)	0.579	<0.001	-	-	0.745	<0.001

Gensini score was significantly associated with cardiovascular risk factors (including FPG, HbA1c and HDL-c), Crouse score, 5-mC and 5-hmC levels. Specifically, the association of 5-hmC and Gensini score (r=0.745, *P*<0.001) was more significant than that of 5-mC (r=0.469, *P*<0.001).

### Diagnostic value of 5-hmC levels and Crouse score for coronary atherosclerosis

In the univariate binary logistic regression analysis (Table 3), the history of diabetes, FPG, HbA1c, hs-CRP, Crouse score, 5-mC and 5-hmC levels were correlated with coronary atherosclerosis. After adjustment for the history of diabetes, FPG and HbA1c, hs-CRP (OR=1.268, 95%CI 1.013–1.588), Crouse score (OR=1.863, 95%CI 1.053–3.297) and 5-hmC (OR=1.767, 95%CI 1.250–2.499) were significantly associated with coronary atherosclerosis, while there was no significant association between 5-mC level and coronary atherosclerosis. With a 0.01% increase in 5-hmC, the risk of coronary atherosclerosis increased by 0.767 times.

**Table 3:** Univariate and multivariate binary logistic regression analysis of cardiovascular risk factors to predict the risk of coronary atherosclerosis.

**Variables**	**Univariate analysis**		**Multivariate analysis**	
	**OR (95% CI)**	***P***	**OR (95% CI)**	***P***
Male/Female	0.481 (0.225–1.030)	0.060		
Age (years)	1.024 (0.953–1.100)	0.511		
Smoker (n)	2.093 (0.096–4.565)	0.063		
History of HP	2.130 (0.961–4.722)	0.063		
History of DM	3.680 (1.645–8.235)	0.002	4.487 (0.227–88.680)	0.324
Stroke	2.489 (0.704–8.798)	0.157		
BMI (Kg/m^2^)	1.048 (0.930–1.182)	0.442		
FPG (mmol/L)	1.782 (1.219–2.605)	0.003	0.972 (0.307–3.071)	0.961
HbA1c (%)	1.785 (1.191–2.674)	0.005	2.861 (0.717–11.422)	0.137
TC (mmol/L)	1.171 (0.913–1.502)	0.214		
LDL-c (mmol/L)	1.383 (0.939–2.036)	0.100		
HDL-c (mmol/L)	0.763 (0.544–1.070)	0.117		
hs-CRP (mg/L)	1.099 (1.020–1.184)	0.014	1.268 (1.013–1.588)	0.038
Crouse score	1.795 (1.445–2.230)	<0.001	1.863 (1.053–3.297)	0.033
5-mC (%)	3.221 (1.851–5.604)	<0.001	0.983 (0.386–2.505)	0.972
5-hmC (%) per 0.01%	1.484 (1.265–1.741)	<0.001	1.767 (1.250–2.499)	0.001

OR = odds ratio; 95% CI = 95% confidence intervals; Odds ratio more than 1 indicates that hs-CRP, Crouse score and 5-hmC are risk factors. The OR value of 5-hmC represents altered risk of coronary atherosclerosis odds per 0.01% change in 5-hmC level.

**Figure 2:**
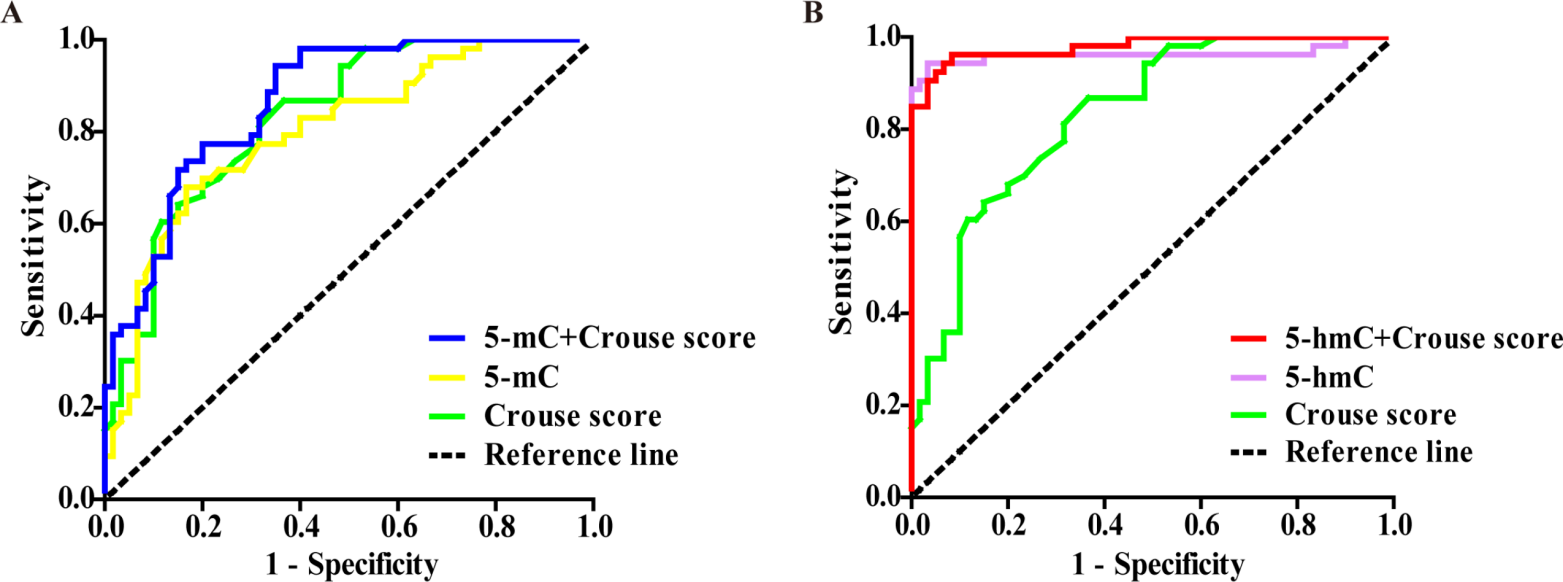
**Comparisons of diagnostic power among 5-mC, 5-hmC, Crouse score and their combination for coronary atherosclerosis by ROC curves.** (**A, B**) The AUC was 0.865 for 5-mC+Crouse score (blue line), 0.800 for 5-mC (yellow line), 0.831 for Crouse score (green line), 0.980 for 5-hmC+ Crouse score (red line) and 0.961 for 5-hmC (purple line).

The diagnostic value of 5-mC, 5-hmC, Crouse score and their combinations for coronary atherosclerosis were identified by receiver operating characteristic (ROC) curves. As shown in Table 4 and Figure 2, 5-mC level, Crouse score and their combinations have similar ROC curves, while the areas under the curves (AUC) of 5-hmC (0.961, 95%CI 0.907–0.988) was larger than that of 5-mC (0.800, 95%CI 0.714–0.869) and Crouse score (0.831, 95%CI 0.749–0.895) for coronary atherosclerosis with significant differences, respectively. The combination of 5-hmC level and Crouse score had the largest AUC (0.980, 95%CI 0.933–0.997), which showed better sensitivity but lower specificity than 5-hmC, but no statistical difference was found.

**Table 4:** The ROC curve parameters of 5-mC, 5-hmC, Crouse score and their combinations for diagnosing coronary atherosclerosis.

	**AUC**	**95% CI**	**Youden index**	cut-off point*	**Sensitivity (%)**	**Specificity (%)**
5-hmC+ Crouse score	0.980	0.933–0.997	0.8789	0.17	96.23	91.67
5-hmC	0.961	0.907–0.988	0.9101	0.12	94.34	96.67
5-mC+ Crouse score	0.865	0.788–0.922	0.5934	4.52	94.34	65.00
Crouse score	0.831	0.749–0.895	0.5013	2.50	86.79	63.33
5-mC	0.800	0.714–0.869	0.5126	3.53	67.92	83.33

^*^Cut-off point were determined according to the ‘Youden index’ derived directly from the ROC curves.

## DISCUSSION

Carotid plaque can predict the future development of coronary atherosclerosis and is a common and robust marker for coronary atherosclerosis [22]. DNA methylation and hydroxymethylation are closely associated with carotid and coronary atherosclerosis. Our previous study [23, 24] showed that DNA methylation and hydroxymethylation levels in elderly patients with myocardial infarction (MI) and coronary heart disease (CHD) were significantly increased and had positive correlation with the degree of coronary atherosclerosis, which was consistent with other reports [10, 25, 26]. Furthermore, our results identified 5-hmC was more closely associated to MI and CHD than 5-mC [23, 24]. Elevated DNA methylation was reported as a biomarker for cardiovascular disease [27], while few study about DNA hydroxymethylation as a biomarker were published. Thus, the aim of the study is to determine whether DNA methylation and hydroxymethylation combined with carotid plaque can be helpful to the diagnosis of coronary atherosclerosis.

Our results clarified that the CAS patients showed higher prevalence of CHD and stroke, higher FPG, CIMT, Crouse score and Gensini score than controls (Table 1). With the increasing of severity of carotid plaque and Crouse score, a stepwise upward trend was observed both in 5-mC and 5-hmC levels (Figure 1), which were significantly correlated with the risk factors such as FPG and HbA1c levels, Crouse score and Gensini score (Table 2). 5-hmC level had a more closely correlation with Gensini score than 5-mC. After adjusting for the risk factors, only 5-hmC, hs-CRP and Crouse score were the risk factors for coronary atherosclerosis and no association was observed between 5-mC level and coronary atherosclerosis (Table 3). ROC analysis indicated that 5-mC, 5-hmC, Crouse score and their combinations for coronary atherosclerosis had the diagnostic value (Table 4 and Figure 2).

Ample evidence has demonstrated DNA methylation and hydroxymethylation were involved in the formation and development of carotid and coronary atherosclerosis [8–10, 27–30], but 5-mC and 5-hmC levels in coronary atherosclerosis are controversial [31]. Our study is believed to be the first time to identify that both DNA methylation and hydroxymethylation levels gradually increase with the increase of Crouse score and the severity of carotid atherosclerosis, and the trend of 5-mC and 5-hmC were consistent with previous studies [10, 25–27, 32].

Carotid atherosclerosis has been confirmed as a window that responds to systemic atherosclerosis including coronary atherosclerosis [13–15]. Spearman correlation analysis showed that 5-hmC level and Crouse score were significantly correlated with Gensini score. Even after adjustment of other risk factors, 5-hmC and Crouse score still correlated with coronary atherosclerosis. With a 0.01% increase in 5-hmC, the risk of coronary atherosclerosis increased by 0.767 times. It was reported that the quantification of bulk levels of 5-hmC is relatively rare, with levels varying by tissue from < 0.1 to 0.7% of cytosines globally [10, 33]. Comparing these variants diagnostic value for coronary atherosclerosis, ROC analysis indicated that 5-hmC level had higher AUC, sensitivity and specificity than 5-mC and Crouse score with significant differences. When 5-hmC level combined with Crouse score, the largest AUC was displayed with 5% of specificity absence, but no difference was found comparing with 5-hmC single. Therefore, 5-hmC combined with Crouse score may be used as a suitable biomarker for coronary atherosclerosis, which is a new discovery compared to previous research [27].

An anterior study reported TET2 somatic mutations in blood cells play a causal role in atherosclerosis and partial bone marrow reconstitution with TET2-deficient cells led to a marked increase in atherosclerotic plaque size [30]. According to the report that approximately 10-20% of the population over age 70 had observable clonal hematopoiesis [34–36], we expected a decrease of TET2 expression, a higher 5-mC level and a lower 5-hmC level in elderly people with significant atherosclerosis. But in our studies, we surprisingly found the levels of 5-mC and 5-hmC and TET2 expression elevated with the increase of the severity of coronary atherosclerosis, while DNMTs expression had no significant change [24]. Except the elevation of 5-hmC, our results were different with a recent report in 5-mC level, DNMTs and TETs expression [10].

Clonal hematopoiesis (CH) is defined as the process that somatic mutations in preleukemic driver genes within hematopoietic stem/progenitor cell (HSPC) confer fitness advantages leading to their clonal amplification [37, 38]. It is reported that somatic mutations most commonly occurred in the genes DNMT3A, TET2 and ASXL1, with each carrying a 1.7 to 2 fold risk for cardiovascular events [34]. DNMTs and TETs are the core genes that determine DNA methylation and hydroxymethylation levels. As the stable DNA methylation and hydroxymethylation products, 5-mC and 5-hmC conveniently reflect the DNMTs and TETs functions, but not the somatic mutation of DNMT3A and TET2 in leukocytes. This is the fundamental cause of the gap between our expectations and results. The comparison of 5-hmC level will be consequent only after whole-exome sequencing in population with different atherosclerosis level. Because of the high costing of sequencing, 5-hmC level is still reasonable to indicate DNA hydroxymethylation level and to associate with coronary atherosclerosis.

Why does 5-hmC have greater diagnosis value than 5-mC? DNA methylation and demethylation both are the dynamic processes. As an intermediate product in DNA demethylation, the 5-hmC is the oxidized product of 5-mC [33]. Though 5-hmC has the relatively low abundance in all cell types, it is a more stable modification of genomic DNA than 5-mC [10, 39]. Additionally, the percentage of 5-hmC in normal human tissues measured is very low in heart (0.05-0.06%) [40]. Unlike the content of 5-mC, which is only 1~2.5 fold different in human tissues, 5-hmC content showed a 13-fold greater difference in different tissues [40]. Our published study found 5-hmC was higher in CHD patients compared with the control subjects [24], but its level was still much lower compare to 5-mC in CHD patients. Because of the low level, 5-hmC became more sensitive than 5-mC, with a very limited change reflecting different disease status. The specific mechanism of 5-hmC acting on coronary atherosclerosis requires further study to confirm.

Our study is a cross-sectional study and has several limitations. The major limitation was that the number of patients in control group was relatively small, in that it was obviously difficult to enroll elderly patients without CAS clinically. A further limitation is that our study is based on CAS patients and the cross validation analysis is needed to determine whether DNA hydroxymethylation combined with carotid plaques can be used to the diagnosis of coronary atherosclerosis in the unselected general population.

In summary, we have demonstrated that 5-mC and 5-hmC levels were significantly increased in CAS patients. It is the first time we confirmed that DNA hydroxymethylation combined with carotid plaques may provide meaningful information for the diagnosis of coronary atherosclerosis.

## METHODS

### Study population

Between January 2018 and May 2018, the study enrolled 113 patients admitted to the First Affiliated Hospital of Chongqing Medical University (Chongqing, China) with 22 controls and 91 CAS patients matched by age and gender. This study was approved by the Human Ethics Committee (NO. 2016-39) and registered on ClinicalTrials (No. NCT03462277), and all study participants provided written informed consent. Carotid ultrasound was used for the diagnosis of CAS. Control group was defined as people with negative findings in carotid ultrasound. CAS patients were defined as plaque formation of at least one lesion in a carotid artery or branches. All patients underwent coronary angiography and CHD patients had severe stenosis of at least one lesion in a coronary artery or branches.

### Crouse score and Gensini score

Crouse score and Gensini score were used to assess the severity of carotid and coronary atherosclerosis. The score of carotid plaques by carotid ultrasound was calculated by Crouse's method: the maximum thickness of isolated atherosclerotic plaques of the ipsilateral carotid artery were added without considering the length of each plaque. The sum of the bilateral carotid plaque scores were total plaque score [41–43]. Gensini score of coronary atherosclerosis by coronary angiography was calculated as follows: the score of a lesion equals the severity coefficient of lesion segment multiplies the score of stenosis degree and final Gensini score of a patient equals the sum scores of all the lesions, as described previously [44, 45].

### Isolation of peripheral blood mononuclear cells (PBMCs) from peripheral blood

PBMCs were obtained by centrifugation of whole blood (∼ 5ml) through mononuclear cells separation solution (Tian Jin Hao Yang Biological Manufacture Co., Ltd., Tianjin, China) at 2050 rpm for 25 min at room temperature; mononuclear cells fraction was taken, washed twice with PBS, washed once with Erythrocyte lysate (Beijing Solarbio Technology Co., Ltd., Beijing, China) and centrifuged at 3000 rpm for 10 min at room temperature. Separated mononuclear cells samples were stored at -80ଌ, which had never been thawed until use.

### Quantification of DNA methylation and hydroxymethylation

Genomic DNA was isolated from PBMCs using Blood Genomic DNA Purification Kit (GMbiolab Co., Ltd., Taiwan, China) and quantified by Nanodrop 2000 (Thermo Fisher Scientific, Waltham, MA, USA). Genomic DNA methylation and hydroxymethylation were determined by measuring 5-mC and 5-hmC using 5-mC DNA ELISA Kit and Quest 5-hmC^TM^ DNA ELISA Kit (Zymo Research, Irvine, CA, USA), respectively [46, 47]. These are the most cited ELISA-based global 5-mC and 5-hmC quantification kit in the literature, and provides scientists with a quick, cost-effective and reliable alternative to chromatographic methods [48, 49]. Assays were performed according to the manufacturer’s instructions loading 100 ng of DNA per well. The absorbance at 405 nm was measured using a Multiskan Spectrum (Thermo Electron Corporation, Waltham, MA, USA).

### Statistical analysis

Data are presented as the mean ± SD, frequency (%) or median (interquartile ranges). Comparisons of baseline characteristics among four groups were made by the Anova test (normally distributed continuous variables), Kruskal-Wallis test (nonnormally distributed continuous variables) and Chi-square test (categorical variables). Bivariate associations were assessed by Spearman’s correlation coefficients. Multivariate binary logistic regression analysis was performed to evaluate the cardiovascular risk factors. To evaluate the diagnostic accuracy of coronary atherosclerosis, ROC plots were constructed and AUCs were calculated for biomarker levels. AUCs were compared according to Delong’s method [50]. Cut-off concentrations were determined according to the ‘Youden index’ derived directly from the ROC curves. All statistical analysis was performed with SPSS software version 22.0 (SPSS Inc., Chicago, USA) and the MedCalc package version 15.2.2 (MedCalc Software bvba, Ostend, Belgium). Two-tailed *P* values <0.05 were considered statistically significant.

### Ethics approval

This study was approved by the Human Ethics Committee (NO.2016-39) and registered on ClinicalTrials (No. NCT03462277), and all study participants provided written informed consent.

## Supplementary Material

Supplementary Table 1
