# Supplementary material for: DNA hydroxymethylation combined with carotid plaques as a novel biomarker for coronary atherosclerosis
Source: Aging (Albany NY). 2019 May 23;11(10):3170–81. doi: 10.18632/aging.101972 (PMC6555448; doi:10.18632/aging.101972)
Supplement: Supplementary Table 1 [file aging-11-101972-s001.pdf]

SUPPLEMENTARY MATERIAL

Supplementary Table 1. Baseline characteristics of control subjects and CHD patients.

	Control (n=60)	CHD (n=53)	*P* value
Male/Female	29/31	35/18	0.058
Age (years)	72.0 (68.3–76.0)	74.0 (69.0–76.0)	0.379
Smoker (n)	17 (28.3%)	24(45.3%)	0.061
CAS (n)	38(63.3%)	53(100%)	<0.001
Stroke (n)	4 (6.7%)	8(15.1%)	0.147
History of HP (n)	34 (56.7%)	39(73.6%)	0.061
History of DM (n)	14 (23.3%)	28(52.8%)	0.001
BMI (Kg/m2)	24.18±3.37	24.66±3.01	0.446
FPG (mmol/L)	5.30 (5.00–5.50)	5.80 (5.45–7.60)	<0.001
HbA1c (%)	5.90 (5.60–6.20)	6.10 (5.75–7.55)	0.010
TC (mmol/L)	3.55 (1.59–4.60)	3.81 (2.61–4.72)	0.334
TG (mmol/L)	1.09 (0.81–1.70)	1.43 (1.10–2.18)	0.003
LDL-c (mmol/L)	2.21±0.97	2.52±1.01	0.099
HDL-c (mmol/L)	1.54 (1.16–2.34)	1.11 (0.93–1.37)	0.001
hs-CRP (mg/L)	1.26 (0.52–2.72)	2.35 (0.59–7.95)	0.043
CIMT (mm)	0.79 (0.70–0.85)	0.80 (0.75–0.90)	0.068
Crouse score	1.65 (0–3.60)	5.10 (3.50–6.75)	<0.001
Gensini score	0	63 (32.25–90.50)	-
5-mC level	3.00 (2.36–3.38)	3.83 (3.34–4.41)	<0.001
5-hmC level	0.06±0.04	0.26±0.09	<0.001
